# Study-protocol for a randomized controlled trial comparing clinical and radiological results after three different posterior decompression techniques for lumbar spinal stenosis: the Spinal Stenosis Trial (SST) (part of the NORDSTEN Study)

**DOI:** 10.1186/s12891-017-1491-7

**Published:** 2017-03-21

**Authors:** Erland Hermansen, Ivar Magne Austevoll, Ulla Kristina Romild, Frode Rekeland, Tore Solberg, Kjersti Storheim, Oliver Grundnes, Jørn Aaen, Jens Ivar Brox, Christian Hellum, Kari Indrekvam

**Affiliations:** 1 Department of Orthopedic Surgery, Ålesund Hospital, Møre and Romsdal Hospital Trust, Ålesund, Norway; 2 Kysthospitalet in Hagevik, Orthopedic Clinic, Haukeland University Hospital, Bergen, Norway; 3 Department of Clinical Medicine, University of Bergen, Bergen, Norway; 4 Department of Research, Levanger Hospital, Nord-Trøndelag Hospital Trust, Levanger, Norway; 5 Department of Neurosurgery, University Hospital of Northern Norway, Tromsø, Norway; 6 Department of Clinical Medicine, University of Tromsø The Arctic University of Norway, Tromsø, Norway; 7 Norwegian National Registry for spine surgery, University Hospital of North Norway, Tromsø, Norway; 8 Communication and Research Unit for Musculoskeletal Disorders (FORMI), Oslo University Hospital and University of Oslo, Oslo, Norway; 9 Department of Orthopedics, Akershus University Hospital, Lørenskog, Norway; 10 Department of Physical Medicine and Rehabilitation, University of Oslo, Oslo, Norway; 11 Department of Orthopaedics, Oslo University Hospital, Oslo, Norway

**Keywords:** Randomized controlled trial, Lumbar spinal stenosis, Surgical treatment, Laminotomy, Osteotomy

## Abstract

**Background:**

There are several posterior decompression techniques for lumbar spinal stenosis (LSS). There is a trend towards performing less invasive surgical procedures, but no multicentre randomized controlled trials have evaluated the relative efficacy of these techniques at short and long-term.

**Method/design:**

A multicentre randomized controlled trial [the Spinal Stenosis Trial (SST) (part of the NORDSTEN study)] including 465 patients aged 18–80 years with neurogenic claudication or radiating pain and MRI findings indicating lumbar spinal stenosis without spondylolisthesis is performed to compare three posterior decompression techniques: unilateral laminotomy with crossover, bilateral laminotomy and spinous process osteotomy. The primary outcome is change in Oswestry Disability Index (ODI 2 years postoperatively). Secondary outcomes are change in EQ-5D, Zurich Claudication Questionnaire, and Numeric Rating Scale for leg-pain and back-pain. Also recorded were Global Perceived Effect score, complications, length of hospital stay, reoperation rate 2 years postoperatively, difference in recurrence of symptoms or postoperative instability, and MRI change in the dural sac area. Further, a 5 and 10 years follow-up is planned with the same outcome measures.

**Discussion:**

Newer and less invasive techniques are increasingly favoured in surgery for LSS. This trial will compare the clinical and radiological results of three different techniques, and may contribute to better clinical decision making in the surgical treatment of LSS.

**Trial registration:**

ClinicalTrials.gov reference: NCT02007083 (November 22, 2013).

## Background

Surgery for lumbar spinal stenosis (LSS) is the most frequent operation performed in the adult lumbar spine [1, 2]. In patients with moderate to severe spinal stenosis, surgery is considered superior to non-surgical treatment for patient reported pain and function. This view is supported by RCT-trials with long-term follow up [3–5] and reviews [6, 7].

This trial focuses on lumbar spinal stenosis without spondylolisthesis.

The choice of surgical decompression technique for patients with LSS is debated. A newly published Cochrane review, conclude that the scientific evidence is of low or very low quality, and that there is no evidence to recommend one particular surgical method over another [6–8]. The trend today is a shift from the more extensive methods, such as a complete laminectomy, to less invasive methods. The rationale is to reduce the surgical trauma, and the risk of surgical complications. Midline retaining surgery is considered beneficial to maintain the bony and ligamentous integrity of the spine. There are several midline retaining methods, which vary by the amount of bony and ligamentous structures that are removed.

The present trial compares three different midline retaining posterior decompression techniques.

The main goal of the surgical procedure is to remove the stenosis in the affected area of the spine. The debate focuses mainly on the extent to which elements outside the spinal canal should be removed and less on the extent of decompression needed [7, 8]. In order to evaluate possible differences between the three different surgical techniques, we will measure the change in MRI dural sac cross-sectional area before and after surgery. We will also study the associations between the change in area and clinical outcome after surgery.

Development or progression of postoperative instability after posterior decompression for lumbar spinal stenosis is still considered as an important complication [9]. Whether one of the three methods is more prone to postoperative instability is also a factor that will be investigated in the present trial.

The NORwegian Degenerative spondylolisthesis and spinal STENosis study (NORDSTEN) study is a multicentre trial consisting of the Spinal Stenosis Trial (SST), the Degenerative Spondylolisthesis Trial (DST) and a observational cohort (OC). This study protocol paper discusses the scientific basis of the Spinal Stenosis Trial (SST).

### AIMS

The specific aims of this study about lumbar spinal stenosis are:

- To compare the clinical outcome after three different posterior decompression techniques: unilateral laminotomy with crossover, bilateral laminotomy and spinous process osteotomy.
- To compare differences in achieved increase of the dural sac cross-sectional area between these three different posterior decompression techniques.
- To explore the association between achieved increase of the dural sac cross-sectional area and clinical outcome after surgery, in order to eventually obtain a threshold value needed for sustainable change in ODI.
- To compare the incidence of postoperative instability after the three different decompression techniques.

## Methods

### Trial design

**Fig. 1:**
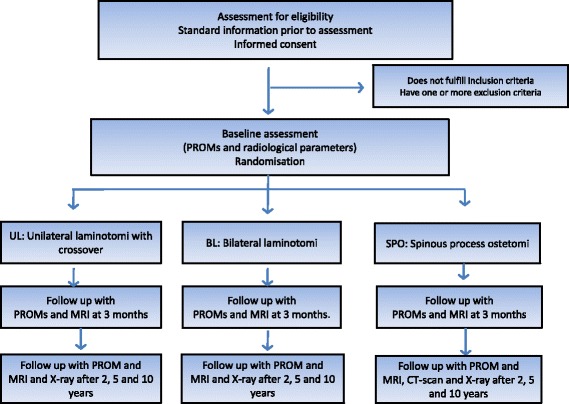
Patient flow through the trial. (PROM: Patient Reported Outcome Measures)

The present study is a multicentre randomized controlled trial. It has a parallel group design, and patients are stratified within each hospital. Follow up will be at 2, 5 and 10 years postoperatively. Eighteen hospitals, orthopaedic and neurosurgical departments, throughout Norway are participating in the study. Further design and follow-up is outlined in the flow-chart (Fig. 1).

### Ethics

Approval for this study has been received from the Norwegian Committees for Medical and Health Research Ethics (2011/2034).

### Patients

Patients, aged 18–80 years, will be recruited from out-patient clinics. All patients with spinal stenosis that are referred for surgical treatment are eligible for inclusion. An orthopedic surgeon or a neurosurgeon will assess clinical symptoms and signs of lumbar spinal stenosis according to the MRI findings and the inclusion and exclusion criteria (listed in Table 1). Eligible patients not included and those excluded from the study will be registered and accounted for.

**Table 1:** Inclusion and exclusion criteria for participating in the surgical trial concerning posterior decompression techniques for lumbar spinal stenosis

Inclusion criteria:
Clinical symptoms of lumbar spinal stenosis: neurogenic claudication or bilateral radiating pain
Not responding to at least 3 months of non-surgical treatment
Radiological findings corresponding to the clinical symptoms: central stenosis, or lateral recess-stenosis.
Able to give informed consent and to answer the questionnaires.
Age > 18 years
Able to understand the Norwegian language, spoken and in writing
Exclusion criteria:
Degenerative lumbar spondylolisthesis, with a slip ≥ 3 mm verified on standing plain x-rays in lateral view.
Not willing to participate in the trial.
Former surgery in the level of stenosis
Fracture, or former fusion in the thoracolumbar region.
Cauda equina syndrom (bowel or bladder dysfunction) or fixed complete motor deficit
ASA- classified 4 or 5.
Age > 80 years
Lumbosacral scoliosis >20°verified on AP-view
Distinct symptoms in one or both of their legs due to other diseases, e.g. polynevropathy, vascular claudication or osteoarthtritis.
Stenosis in > 3e levels
Not able to comply fully with the protocol, including treatment, follow-up or study procedures (psychosocially, mentally and physical).
Participating in another clinical trial that may interfere with the present trial

The CONSORT check list for reporting randomized controlled trials will be followed [10], and the patient flow through the trial is accounted for in Fig. 1. 

### Participating hospitals

Following hospitals in Norway are participating in the study and including patients to the trial: Stavanger University Hospital, Arendal Hospital, Kristiansand Hospital, Skien Hospital, Drammen Hospital, Martina Hansens Hospital, Bærum Hospital, Oslo University Hospital - Ullevål, Akershus University Hospital, Gjøvik Hospital, Elverum Hospital, Lillehammer Hospital, Kysthospitalet in Hagevik, Haukeland University Hospital, Ålesund Hospital, Kristiansund Hospital, Levanger Hospital and University Hospital of Northern Norway.

### Baseline data

Baseline data include demographic data, data concerning comorbidity, radiological classifications and evaluations, American Society of Anaesthesiologists (ASA) grade, and patient reported outcome variables including the Oswestry Disability Index (ODI), Zurich Claudication Questionnaire (ZCQ), EuroQol (EQ-5D), HSCL 25, Numeric Rating Scale for low back pain and leg pain (NRS).

### Randomisation procedure

Eligible patients are randomised into one of three arms, unilateral laminotomy with crossover, bilateral laminotomy and spinous process osteotomy with a 1:1:1 allocation. A randomized block design, stratified by hospital and the blocks made as small as possible, is used to ensure equal distribution of all three treatments. Randomisation is performed in the 6 weeks before surgery. The randomisation procedure is concealed and administered by a study coordination centre at a university hospital, and communicated to a local research coordinator who is not involved in the treatment of the patients. Randomisation is performed after the patient has signed the informed consent form. The result of the randomisation is documented in the patient record. Hence, neither the patients nor the surgeons can influence the type of intervention.

### Interventions

Eighteen hospitals in Norway are participating in the trial, and are recruiting patients. All surgeons are familiar with the different operation techniques. Both through joined gatherings in the operation theater and though a surgical protocol.

The actual level is confirmed by fluoroscope peroperatively. Unilateral laminotomy with crossover and spinous process osteotomy involve a unilateral detachment of the multifidus muscles, whereas this in bilateral laminotomy is done bilaterally. When performing unilateral laminotomy with crossover, loupe magnification or microscope is mandatory, in bilateral laminotomy and spinous process osteotomy loupe magnification or microscope can be used depending on the surgeon’s preference.

### Group A) unilateral laminotomy with crossover (UL)

The laminotomy is first performed ipsilaterally. The decompression of the spinal canal is initiated by a flavectomy followed by a laminotomy of the lower part of the superior lamina, and the upper part of the inferior lamina. Laterally, a medial facetectomy is performed and the patient is then slightly rotated in order to visualize the contralateral side. The dura is retracted, and the decompression is performed contralaterally.

### Group B) bilateral laminotomy (BL)

The decompression of the spinal canal is initiated by a bilateral flavectomy followed by a bilateral laminotomy of the lower part of the superior lamina, and the upper part of the inferior lamina. Laterally, a medial facetectomy is performed.

### Group C) spinous process osteotomy (SPO)

An osteotomy is performed, at the base of the spinous process above (and sometimes under) the affected level. The spinous process is retracted to the contralateral side with intact supraspinal and interspinal ligaments, giving a midline access to the spinal canal. The decompression is first performed in the midline, then laterally at both sides. A laminotomy of the lower part of the superior lamina and the upper part of the inferior lamina is performed, followed by a medial facetectomy. Both nerve roots are visualized, and the lateral recesses are decompressed. Special attention is warranted when a multilevel decompression is performed in order to retain at least 1/3 of the lamina.

We consider it important to visualize the respective medial borders of the pedicles and the nerve roots, from the beginning of the thecal sac passing the pedicle.

Postoperative care will be conducted as usual at the respective hospitals. This includes early mobilization aided by a physiotherapist, but thereafter no specific or structured program of physiotherapy.

### Outcome assessment

In each hospital, non-blinded coordinators (not surgeons) will ensure that the questionnaires are completed at baseline, 3, 12 months, 2, 5 and 10 years. The primary endpoint is 2 years, secondary endpoints at 5 and 10 years.

### Patient reported outcome measures (PROM)

#### Primary outcome

The primary outcome is change from baseline to follow-up in the Oswestry Disability Index (ODI) Norwegian version 2.0 [11]. The ODI includes 10 questions about pain and activities of daily living. Each item has five response categories from no pain related disability (0) to the worst possible pain related disability (5). Responses are transformed into an index ranging from no disability (0) to the worst possible disability (100). The ODI questionnaire is the most widely used and validated outcome measure in spinal surgery [12, 13].

#### Secondary outcomes

Secondary patient reported outcomes are changes from baseline to follow-up in the EuroQol 5-dimensional questionnaire utility index (EQ-5D), the Zurich Claudication Questionnaire (ZCQ-score), a ten point Numeric Rating Scale (NRS) for low back pain and for leg-pain, and a global perceived effect scale.

The EQ-5D is a generic measure of health-related quality of life. Five domains are rated: mobility, self-care, activity, pain and anxiety, each by three response categories to provide a utility index ranging from −0.59 (worst possible) to 1.0 (best possible). In addition EQ-VAS provides a single score of the patient’s health condition. EQ-5D is validated for the Norwegian population. Despite its large measurement error it is often used in research for spinal conditions [14, 15]. The ZCQ is a disease specific questionnaire for lumbar spinal stenosis [16]. It includes symptom severity, physical activity and patient satisfaction during follow-up. The global perceived effect scale is a seven point scale, which is recommended for clinical trials of chronic pain conditions [17]. It has six response categories: 1 = completely recovered, 2 = much improved, 3 = slightly improved, 4 = no change, 5 = slightly worse, 6 = much worse and 7 = worse than ever. All questionnaires are validated for lumbar spinal stenosis patients [15, 16, 18] and are in close accordance with recommended PROMs for the study of low back pain conditions [19].

We will also compare the proportion of patients classified as success, between the groups. Based on change in ODI score after the operation, the patients will be dichotomized into success and non-success groups. A success is defined as a patient with an improvement in ODI score of at least 30%. This value is based on a national register study from the Norwegian Registry for Spine Surgery (NORSpine), and is also in accordance with recommendations from the IMMPACT group, when comparing clinical effect between groups [20].

The local study coordinator will record complications and adverse events, length of hospital stay, duration of surgery, blood loss and the need for blood transfusion.

### Radiological evaluations

Radiological evaluations will be performed by independent investigators.

Preoperative and 3 months postoperative MRI scans will be evaluated to measure the extent of decompression by calculating the change in dural sac cross-sectional area at the most stenotic level (square millimetres [21]. The association between the increase in dural sac cross-sectional area and clinical improvement (primary outcome) will be assessed. We will compare the increase in dural sac cross-sectional area in patients who achieve a minimal clinically important change of the ODI with those who do not.

To assess postoperative instability, erect radiographs of the lumbosacral column with functional images are taken preoperatively. Then erect images (without functional images) are taken after 2, 5 and 10 years. Instability will be noted as an increase (in mm) after the method described by Dupuis et al. [22].

### Sample size

The trial is conducted with a superiority design. The study is designed to detect a difference of 7 ODI-points between the groups. With a standard deviation of 18, a significance level of 0.02, 80% power and a drop-out rate of 15%, we need to include 155 patients in each group. Thus, we plan to include 465 patients over a 4–5 year period. The analysis will be performed according to the intention to treat principles. If the number of drop-out or missing data exceeds 15%, imputation will be performed.

### Statistics

All statistical evaluations will be performed by a statistician blinded to the treatment given.

Comparing three methods increases the risk of statistical error, and therefore, we lowered the significance level from 5 to 2%. Since this is a randomized controlled trial, we will not adjust for any differences in the baseline characteristics.

We will perform Generalized Linear Models for repeated measures when analyzing groups of related dependent variables that represent different measurements of the same attribute. The mean ODI change in each group after 2, 5 and 10 years will be compared between the groups.

Predictor analysis will be performed to investigate whether the following factors predict good results after surgery: treatment group, age, sex, BMI, smoking, preoperative dural sac cross-sectional area (in mm^2^), percentage achieved decompression, preoperative ODI-score and preoperative NRS-score for leg pain and back pain.

## Discussion

The objective of the current trial is to study the long term clinical and radiological results of three different posterior decompression techniques. We will also examine whether the extent of decompression influence the clinical results, and if some techniques give higher risk for postoperatively instability.

We chose a pragmatic design with a limited number of exclusion and inclusion criteria to improve the external validity. Additionally, the surgical methods studied are used in daily clinical practice in Norway.

Ideally, laminectomy should have been included as one of the different surgical methods in this RCT, as laminectomy is still considered by many to be a “gold standard” [8]. In Norway, laminectomy for LSS is rarely performed and the hospitals were therefore not willing to participate in a trial using laminectomy as one of the surgical methods. Spinous process osteotomy also has a midline access, and may be regarded as a proxy for laminectomy. Many of the arguments for laminectomy (visibility, access to the lateral recesses and achieving a wide decompression) are used to advocate for spinous process osteotmy.

The NORDSTEN study, spinal stenosis trial, is is pragmatic in design and performed using “usual care” and surgical techniques used in Norway today.

There is disagreement about the extent to which the dural sac compression is related to the clinical symptoms of lumbar spinal stenosis [23, 24] and whether the extent of decompression after surgery for LSS influences clinical outcomes [25, 26]. We will examine the increase in dural sac area obtained postoperatively after performing the three surgical methods of decompression. We hypothesise that the increase in dural sac area correlates with improvements in clinical symptoms, and that there is a minimal increase in area that is needed to give long-term relief of symptoms.

There were several statistical challenges we had to consider when drafting the outline of this trial.

First, comparing three methods increases the risk of statistical error. Therefore, we lowered the significance level from 5 to 2%. Instead of a 5% risk statistical type I error we have 6% risk of statistical error. Significance level for Post Hoc testing will be adjusted accordingly.

Second, the trial is planned with statistical superiority design. A superiority design is in our opinion best suited when none of the three posterior decompression techniques are considered as gold standard [8]. The statistical power analysis was performed to reveal a statistical difference in mean ODI of 7 ODI points between the groups. The Minimally Clinically Important Difference (MCID) for ODI, on an individual basis is in some studies reported to lie between 8 and 12 points [27–29]. However, a minimal clinical relevant difference between groups is not scientifically discussed. Mead et al. is proposing a difference in mean ODI between groups of 4 points as relevant [30]. But, so far as we know, there is no consensus in the literature of a certain value comparing mean ODI in groups. As a supplement to comparing mean in the three groups we will dichotomize the patients into responders and no-responders. This will able us to analyse the primary outcome more thoroughly [30].

Results will be disseminated via publications in general journals and in more specific spine and imaging journals and at conferences.

This RCT will provide insight into the long-term clinical results of the three posterior decompression techniques, and may provide us with parameters which can predict preference for one of the three methods. Up till now (November-2016) we have included over 320 patients throughout Norway. We anticipate another year of recruitment.

## Abbreviations

EQ-5D: EuroQol 5-dimensional questionnaire utility index
GPE: Global perceived effect- scale
LSS: Lumbar sinal stenosis
NRS: Numeric rating scale
ODI: Oswestry disability index
ZCQ: Zurich claudication questionnaire -score
